# When time worsens framing: a longitudinal analysis of the psychological effects of the COVID-19 pandemic in women with an eating disorder and their healthy sisters

**DOI:** 10.1007/s00737-023-01398-x

**Published:** 2023-11-15

**Authors:** Paolo Meneguzzo, Enrico Ceccato, Alessandra Sala, Paolo Santonastaso

**Affiliations:** 1 Department of Neuroscience, University of Padova, Via Giustiniani 2, 35128 Padova, Italy; 2 Padova Neuroscience Center, University of Padova, Padova, Italy; 3 Vicenza Eating Disorders Center, Mental Health Department, Azienda ULSS8 “Berica”, Vicenza, Italy

**Keywords:** Eating disorder, COVID-19, Trauma, Longitudinal, Frame bias, Negative frame

## Abstract

The COVID-19 pandemic has profoundly affected individuals with eating disorders (ED), leading to an exacerbation of symptoms worldwide in 2020. However, there is a lack of longitudinal analyses of the psychological burdens experienced by this population. This study aims to longitudinally assess the psychological effects of the COVID-19 pandemic in people with ED and their healthy sisters (HS) 1 and 2 years after the onset of the crisis. A sample of 148 individuals, consisting of 73 with ED and 45 HS, was evaluated in spring 2021 and spring 2022 regarding their current psychological and behavioral states. Participants were also asked to reflect on their feelings and behaviors during the 2020 lockdown. General psychopathology, eating disorders, and trauma-related symptoms were evaluated using validated questionnaires. Both groups showed an overall improvement in psychopathological symptoms with time. Individuals with ED exhibited greater improvement compared to their HS, which may be attributed to their initially higher burden. Individuals with ED reported a negative reframe, characterized by internalizing negative emotions and behaviors related to the 2020 lockdown. This longitudinal evaluation revealed two distinct and contrasting effects. Both ED patients and their HS demonstrated psychological improvement over time. However, people with ED experienced a negative reframe that affected their memory of specific life events, subsequently affecting their psychological well-being. These findings shed light on the clinical severity observed in people with ED during these pandemic years.

## Introduction

According to the transdiagnostic model, eating disorders (EDs) are a set of severe psychiatric conditions characterized by disruptions in eating behaviors and associated thoughts and emotions, often linked to concerns about body size, weight, or shape (Schaumberg et al. 2021). EDs are more prevalent among women (3.8%) than males (1.5%), with the highest incidence occurring during adolescence and posing a significant social burden (Favaro et al. 2019; Swanson et al. 2011). A specific increase in this burden has been recorded during the recent COVID-19 pandemic (Zipfel et al. 2022).

The effects of the COVID-19 pandemic on ED individuals have been recorded worldwide, but the reasons are not yet clear (Haghshomar et al. 2022). The current literature has highlighted the deterioration of psychological well-being in the general population and individuals with ED, with a specific increase especially in adolescent females (Cooper et al. 2022; Devoe et al. 2022; Taquet et al. 2022). Several possible explanations for this phenomenon have been proposed, which seem specific to time, age, and diagnosis (Devoe et al. 2022). The elements most investigated include routine change, restrictions in food access, restriction of healthcare facilities, social isolation, and specific confinement (Gao et al. 2022; Giel et al. 2021; Miniati et al. 2021; Monteleone et al. 2021b). However, recent systematic reviews of the literature related to COVID-19 have shown the presence of several limitations that affected the value of this data in helping experts understand the situation and highlighted the need for more longitudinal studies (Linardon et al. 2022; Schneider et al. 2022).

Preliminary data have suggested the possible role of individuals’ abilities to respond to stressors as a specific factor that could explain what has been found (Monteleone 2021), describing what is happening in the field of EDs as a model for post-traumatic responses. Preliminary evaluations have suggested an overall improvement, rather than a general worsening, of eating symptoms and body image dissatisfaction after an initial severe degradation during COVID-19 lockdowns. This pattern has been observed in the general population and individuals with ED (Robinson et al. 2022; Sharpe et al. 2022). Clinical evidence has highlighted the presence of an interaction between trauma, the COVID-19 pandemic, and treatment outcomes, underlining the roles of isolation, fear of the unknown, illness anxiety, financial hardships, and other negative experiences that could act as comorbidities (Cook et al. 2022). It seems possible to differentiate between an initial period characterized by uncertainty and rapid changes that exacerbated symptomatology and a second period characterized by a decrease in impact (Termorshuizen et al. 2020). A possible explanation could be the presence of an adaptation to the changed environment (Sharpe et al. 2022), even if the situation has not returned to the pre-pandemic level (Milliren et al. 2023). The continued high presentation of EDs may be associated with a long-term traumatic effect, as authors defined this prolonged exposure to the COVID-19 pandemic (Manchia et al. 2022). Indeed, the existing literature has already demonstrated that individuals with a history of trauma and an ED exhibit altered responses to stressors (Meneguzzo et al. 2022b), which may also be connected to certain cognitive aspects. Indeed, traumatic events often require a positive reframing of the experience to mitigate its long-term impact (Munroe et al. 2022). In other words, reframing is a way the brain identifies patterns in chaos and assigns meaning to seemingly meaningless events. When negative reframing is primarily associated with traumatic experiences, it can result in persistent trauma and distress. Failure to achieve a positive reframing can lead to long-term negative effects, including depression, anxiety, and the persistence of post-traumatic stress symptoms (Wong and Yeung 2017). This aspect could serve as one possible explanation for the difficulties in reducing the volume of people seeking help for EDs, which has not yet returned to pre-pandemic levels (Milliren et al. 2023). However, most studies reported data only at the beginning of the pandemic, which excluded conclusions about results, changes, and specific needs of underrepresented populations (Schneider et al. 2022).

One of these underrepresented populations consists of individuals with EDs, who require longitudinal studies to assess the factors that contribute to the burden related to the pandemic. In this context, an intriguing group for comparison with individuals with EDs is their healthy sisters (HSs). These sisters are characterized by specific psychological, biological, and environmental vulnerabilities to EDs (Maon et al. 2020), and they were exposed to the same conditions, particularly those related to the worsening of symptoms during the pandemic (Monteleone et al. 2021a). In fact, the literature has shown the presence of specific effects of the COVID-19 crisis on post-traumatic symptoms linked to interpersonal sensitivity and obsessive-compulsiveness that have differentiated EDs from the general population (Meneguzzo et al. 2022c), but also the presence of a nonspecific interaction between post-traumatic symptoms and psychopathology in people with an ED that requires more focused studies (Meneguzzo et al. 2022a). Looking at two populations that share environmental, psychological, and biological characteristics might help to expand the knowledge about the interaction between environmental changes and eating psychopathology and reduce uncertainty about the ambivalent effects of the COVID-19 crisis. For these reasons, a longitudinal approach to psychopathological changes in people with ED and their sisters could help evaluate the real effects of lockdown and prolonged pandemic.

Therefore, this study aims to assess temporal changes in post-traumatic symptoms and psychopathology among patients with ED and their HS, considering possible variations over time. Our main hypothesis is that all participants will show an improvement in symptomatology over time, with a specific lockdown effect on ED. Additionally, we aim to identify specific factors that could enhance scientific comprehension of the effects of the COVID-19 crisis on the ED population, thereby informing future preventive strategies.

## Materials and methods

### Participants

Women with ED and their HS who participated in the previous study on the psychological effects of the COVID-19 pandemic (Meneguzzo et al. 2022a) were contacted after 1 year for a second evaluation. The patients were referred to the EDs Unit of Vicenza Hospital (Italy), a public healthcare service specializing in EDs serving a population of more than 500,000 people in the northeast of Italy. The Unit offers specialized multidisciplinary treatments based on cognitive behavior therapy, with various levels of care, outpatient, day hospital, or inpatient—tailored to the severity of the disorder. Participants in the ED group were engaged in treatment at T1 and all patients were still in treatment at T2.

The first evaluation of our study (T1) was carried out between January and June 2021, 1 year after the beginning of the COVID-19 crisis, while the second evaluation (T2) was carried out between January and June 2022, 2 years after the national 2020 lockdown. The inclusion criteria were that the participants had to be between 14 and 40 years of age (the usual range of ages of patients treated in the ED unit) and had no history of psychotic symptoms or serious medical conditions. ED patients met the DSM-5 criteria for EDs as evaluated by a trained psychiatrist. HS participants were included with the same age criteria, were co-habitants with the patients, and were screened for the exclusion criteria of a personal history of any ED or psychiatric condition through clinical interviews with a trained psychiatrist.

Participation in both study waves was voluntary and did not affect the trajectory of treatment. Data collection was carried out using an online survey (www.surveymonkey.com). A direct link to survey was provided to the participants after they agreed to participate. The local Ethics Committee approved the study design in accordance with the Declaration of Helsinki. All participants signed informed consent forms.

### Questionnaires

The same questionnaires applied in the first wave—the brief symptoms checklist (SCL-58), the ED examination questionnaire (EDE-Q), and the impact of events scale (IES-R)—were used in the second evaluation.

The SCL-58 is a widely used self-report questionnaire derived from the SCL-90R. It comprises 58 items and assesses general psychiatric symptoms and psychological distress (Derogatis et al. 1974). It provides scores with a global severity index (GSI, Cronbach’s *α* = 0.976) and five subscales: somatization (SOM), obsessive-compulsiveness (OBC), interpersonal sensitivity (IPS), depression (D), and anxiety (A). Each item is rated on a 5-point scale; higher total scores reflect greater symptomatology.

The EDE-Q is a validated 28-item self-report questionnaire structured to evaluate eating symptomatology and concerns (Fairburn and Beglin 1994). It contains four subscales—restraint eating, eating concern, shape concern, and weight concern—and a total score (Cronbach’s *α* = 0.922). Higher scores reflect greater eating-related pathology.

IES-R is a validated 22-item self-report questionnaire that assesses subjective distress related to a specific event. It has been widely used in the years since the COVID-19 pandemic (Forte et al. 2020). It comprises three subscales—avoidance, intrusion, and hyperarousal—and a total score (Cronbach’s *α* = 0.954). The cut-off point for clinical relevance for the impact of an event is 24.

Finally, a specific scale was proposed at both time points to assess the psychological and behavioral effects of the pandemic and related confinements. At both times, we asked the participants to report on their experiences during the national lockdown between March and April 2020 and their current experiences. The questionnaire was validated in a previous international study focused specifically on the psychological and behavioral effects of the pandemic: Section II of the COVID isolation eating scale (Fernández-Aranda et al. 2020). Therefore, we obtained two different evaluations: lockdown (Cronbach’s *α* = 0.817) and current effect (Cronbach’s *α* = 0.805). Responses were collected on a 6-point Likert scale (0 = never, 5 = always). The questionnaire comprised nine items: body concerns, food restriction, weight change, binging, exercise, laxative use, diuretics, purging, and body checks. The total score was used to evaluate the reframing effect by comparing the scores between T1 and T2.

### Statistical analysis

We assessed baseline differences between responders at T2 and nonresponders at T2 using the Mann–Whitney tests for both ED and HS subgroups separately. For categorical data, we applied a chi-square test.

Subsequently, we considered exclusively individuals who responded at both T1 and T2. We evaluated differences between T1 and T2 for the ED and HS subgroups separately using the Wilcoxon test, considering the nonparametric distribution of most of the variables included in the study. The effect size for the Wilcoxon test (*r*) was calculated as *r* = *Z*/√*N* and was interpreted as small if *r* < 0.1, medium if 0.1 < *r* < 0.5, and large if *r* > 0.5. A mixed-effect model approach has been used to evaluate the possible effect of time over psychopathology and COVID-19 concerns, due to the unbalanced nature of the data (Cnaan et al. 1997). Linear regression analyses were used to evaluate the possible role of psychological variables evaluated at baseline (T1) at T2. This was done to look for factors that might predict recollections of the event. All analyses were performed with IBM SPSS Statistics version 25.0. For all analyzes, the alpha was set at *p* < 0.05.

## Results

**Fig. 1:**
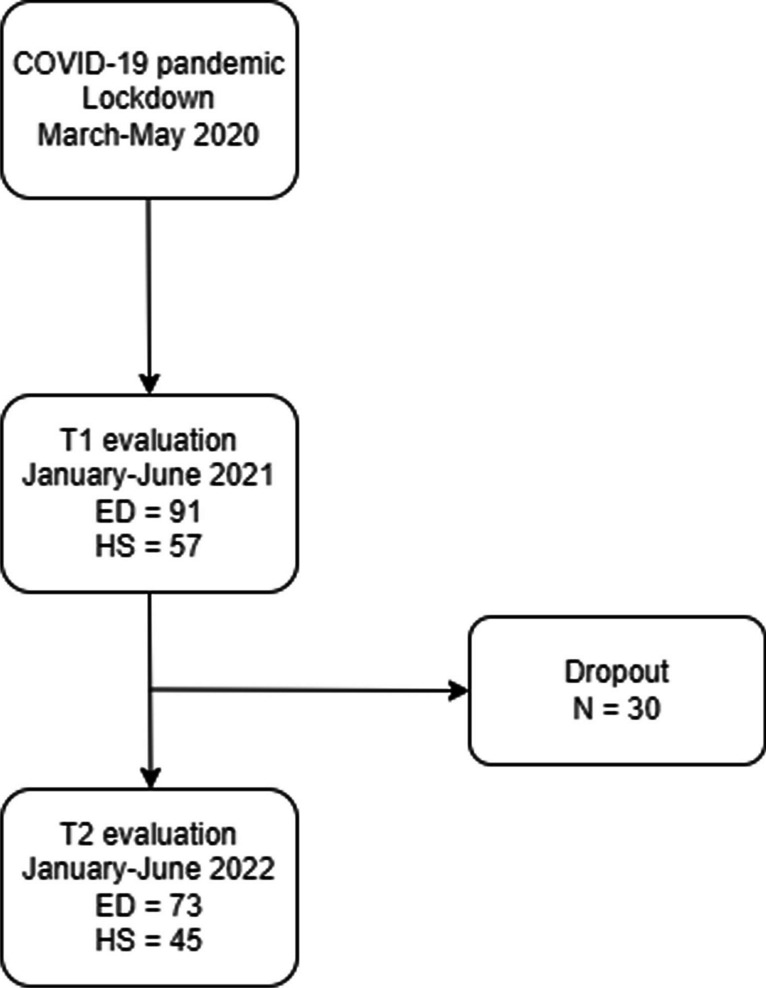
Flow chart of the study showing enrollment in T1 (2021), and responders and nonresponders at T2 (2022)

A total of 148 subjects, including ED patients and their HSs, participated in the T1 study (Meneguzzo et al. 2022c). Of these, 30 (20.3%) participants withdrew from the study at T2 by not responding to the email with the survey link, reducing the sample of participants who completed the evaluation to 118 women. Looking at the participants in this study, 81.2% (*N* = 73) of the original participants of the ED group participated in the T2 evaluation, while 78.9% (*N* = 45) of the HS participated in the T2 evaluation (see Fig. 1 for a flow chart of the study). There were no differences in the response rate between the groups (*Χ*^2^ (1, *N* = 148) = 0.035, *p* = 0.851), or comparing EDQ-Q global score at T1 (*t*(143) = 0.011, *p* = 0.991), SCL-58 GSI (*t*(137) = 0.830, *p* = 0.408), and IES-R Total (*t*(135) = 0.003, *p* = 0.998) between responders and nonresponders. For these longitudinal analyzes, we included only people who participated in both T1 and T2.

**Fig. 2:**
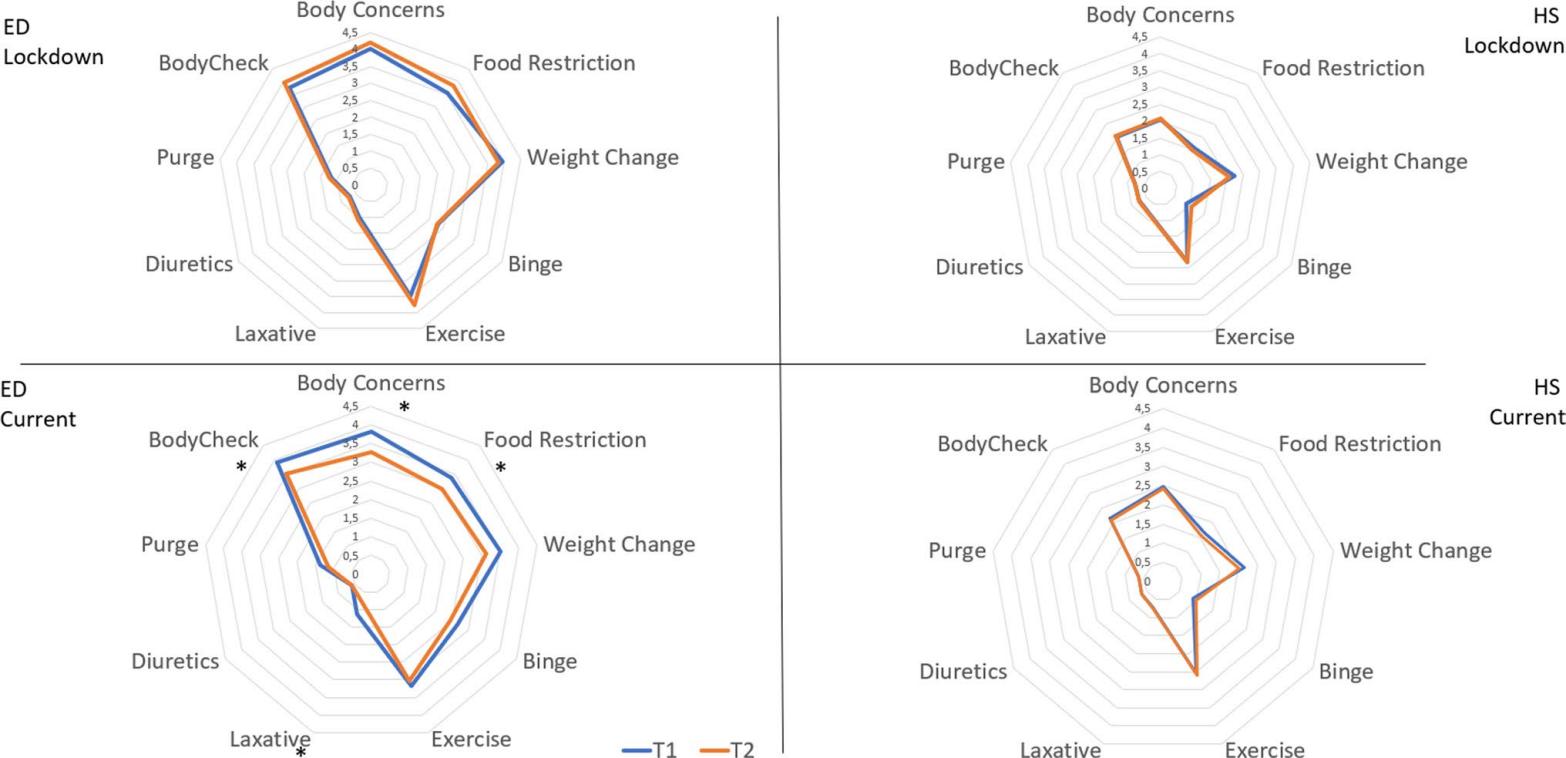
Graphical representation of psychological concerns evaluated after 1 year (T1) and 2 years (T2) from the beginning of the COVID-19 pandemic using the COVID isolation eating scale. Constructs characterized by significant changes between time points are marked with a *

The ED group consisted of 40 women with anorexia nervosa, 20 with bulimia nervosa, five with binge eating disorder, and eight with an ED otherwise specified. The mean age of the ED group was 21.94 ± 6.58 years, with a mean BMI of 20.90 ± 4.90 kg/m^2^. The 45 HS had a mean age of 21.25 ± 2.72 years and a mean BMI of 22.30 ± 5.07 kg/m^2^. No differences emerge in age (*t*(114) = 0.306, *p* = 0.760) or BMI between groups (*t*(107) = 0.487, *p* = 0.621). Comparisons of psychological aspects are reported in Table 1. All psychopathological constructs evaluated showed reductions in severity in both groups. For the HS group, changes with large effect sizes were found for weight concerns (*r* = 0.33) and hyperarousal (*r* = 0.32). For the ED group, a large effect size was found for hyperarousal (*r* = 0.31), small effect sizes were found for somatization (*r* = 0.24), eating concern (*r* = 0.28), shape concern (*r* = 0.24), and total score of IES-R (*r* = 0.29) (see Fig. 2 for a graphical representation). In the linear mixed model analysis, there was a significant main interaction time by group for D (*F*(1,95) = 4.193, *p* = 0.043), GSI (*F*(1,95) = 3.774, *p* = 0.049), hyperarousal (*F*(1,92) = 8.689, *p* = 0.004), IERS total score (*F*(1,91) = 5.281, *p* = 0.024), eating concern (*F*(1,91) = 4.237, *p* = 0.042), and shape concern (*F*(1,3.748) = 3.848, *p* = 0.048), while there was no significant interaction time by group for SOM (*F*(1,95) = 3.575, *p* = 0.062), OBC (*F*(1,95) = 2.840, *p* = 0.095), IPS (*F*(1,94) = 2.911, *p* = 0.091), A (*F*(1,94) = 2.642, *p* = 0.107), avoidance (*F*(1,92) = 1.322, *p* = 0.253), intrusion (*F*(1,92) = 2.561, *p* = 0.113), restraint (*F*(1,95) = 2.168, *p* = 0.144), weight concern (*F*(1,92) = 0.001, *p* = 0.979), and EDE-Q global score (*F*(1,90) = 2.101, *p* = 0.151).

**Table 1:** Psychological evaluation of the participants who responded to the questionnaire in both Spring 2021 and Spring 2022

	ED, *n* = 73			HS, *n* = 45		
	T1	T2	*Z* *p*	T2	T2	*Z* *p*
SCL-58						
SOM	1.81 (0.80)	1.58 (0.86)	− 2.081 .037	0.90 (0.69)	0.91 (0.68)	− .135 .892
OC	1.77 (1.02)	1.53 (0.98)	− 1.631 .103	0.94 (0.72)	0.95 (0.70)	− .106 .915
IS	1.65 (0.98)	1.39 (0.90)	− 1.656 .098	0.86 (0.80)	0.89 (0.79)	− .509 .611
D	1.87 (0.82)	1.65 (0.82)	− 1.571 .116	0.92 (0.72)	0.97 (0.70)	− 1.160 .246
A	1.89 (0.90)	1.68 (0.87)	− 1.664 .096	0.91 (0.67)	0.93 (0.67)	− 1.029 .303
GSI	1.88 (0.83)	1.64 (0.85)	− 1.682 .093	0.93 (0.67)	0.95 (0.66)	− .509 .611
EDE-Q						
Restraint	3.08 (1.70)	2.68 (1.82)	− 1.600 .110	0.46 (0.91)	0.48 (0.92)	< .001 1.000
Eating concern	3.32 (1.23)	2.93 (1.74)	− 2.370 .018	0.67 (1.13)	0.69 (1.14)	− .141 .888
Shape concern	4.71 (1.20)	4.23 (1.79)	− 2.021 .043	1.54 (1.53)	1.55 (1.55)	− .105 .916
Weight concern	3.77 (1.56)	3.75 (1.89)	− .672 .501	1.21 (1.34)	1.13 (1.37)	− 2.232 .026
Global	3.70 (1.19)	3.40 (1.63)	− 1.728 .084	0.97 (1.14)	0.96 (1.16)	− .527 .598
IES-R						
Avoidance	1.39 (0.90)	1.30 (0.99)	− 1.188 .235	1.14 (0.77)	1.19 (0.79)	− .512 .609
Intrusion	1.16 (0.88)	1.06 (0.97)	− 1.640 .101	0.77 (0.65)	0.82 (0.66)	− 1.581 .114
Hyperarousal	1.64 (0.96)	1.35 (1.03)	− 2.668 .008	1.11 (0.77)	1.05 (0.75)	− 2.136 .033
IES-R total	1.43 (0.99)	1.23 (0.92)	− 2.483 .013	0.98 (0.68)	1.04 (0.68)	− .949 .343

The table reported means and (standard deviations). *Z*: Wilcoxon test

*ED*, patients with an eating disorder; *HS*, healthy sister. *SOM*, somatization; *OC*, obsessive–compulsive; *IS*, interpersonal sensitivity; *D*, depression; *A*, anxiety; *GSI*, global severity index; *IES-R*, impact of event scale; *EDE-Q*, eating disorder examination questionnaire; *SCL-58*, symptom checklist

Regarding concerns of the COVID-19 pandemic effects, significant changes were found only in the ED group. There was a reduction in concern between T1 and T2 (Spring 2021 vs. Spring 2022), but an increase in concern recalling the first COVID-19 lockdown (Spring 2020) over time (see Table 2 for details). The mixed-effects model analyzes confirmed the presence of significant interaction time by group for both current (*F*(1,103) = 4.026, *p* = 0.047) and lockdown concerns (*F*(1,110) = 3.998, *p* = 0.040).

**Table 2:** Evaluation of the effects of COVID-19 pandemic on the participants, section II of the COVID isolation eating scale

	ED			HS		
	T1	T2	*Z* *p* (*r*)	T1	T2	*Z* *p* (*r*)
Lockdown effects	23.90 (8.11)	26.00 (6.70)	− 2.272 .023 (.26)	13.38 (10.34)	13.40 (10.73)	− .170 .865 (.02)
Current effects	23.58 (7.00)	20.85 (8.27)	− 3.016 .003 (.35)	13.95 (9.54)	13.71 (9.72)	− .595 .552 (.09)

The table reported means and (standard deviations). *Z*: Wilcoxon test. *r*: effect size for the Wilcoxon test

*ED*: patients with an eating disorder; *HS*: healthy sister

Finally, looking at the effects of the relationships between COVID-19 concerns in T2 and psychopathology as recorded in T1, there was a predictive role of avoidance in the psychopathology of the ED only for individuals with an ED (*R*^2^ = 0.391, *F*(6,47) = 5.024, *p* < 0.001). Meanwhile, in HS, the effects of COVID-19 in T2 were predicted only by the effects in T1 (*R*^2^ = 0.739, *F*(6,35) = 20.379, *p* < 0.001) (see Table 3 for details).

**Table 3:** Regression analysis for COVID-19 concerns in T2

	ED group				HS group			
	*B*	*SE B*	*β*	*t* (*p*)	*B*	*SE B*	*β*	*t* (*p*)
GSI	− 0.191	1.819	− 0.020	− 0.107 (.915)	− 1.770	1.852	− 0.124	− 0.957 (.346)
EDE-Q Global	2.877	1.067	0.412	2.696 (.010)	0.053	0.767	0.006	0.069 (.945)
Avoidance	4.239	1.650	0.445	2.569 (.013)	2.425	1.780	0.344	1.487 (0.101)
Intrusion	− 2.871	2.185	− 0.298	− 1.314 (.195)	− 0.541	2.829	− 0.036	− 0.191 (.849)
Hyperarousal	1.277	2.009	0.145	0.636 (.528)	− 1.701	2.634	− 0.137	− 0.646 (.523)
Current effects	0.097	0.161	0.082	0.602 (.550)	0.808	0.086	0.797	9.381 (< .001)

*ED*, patients with an eating disorder; *HS*, healthy sister; *GSI*, global severity index; *EDE-Q*, eating disorder examination questionnaire

## Discussion and conclusions

This longitudinal study involving individuals with ED and their HS indicates an overall improvement in general and specific psychopathology in both groups over a 1-year time period (from Spring 2021 to Spring 2022), accompanied by emotional detachment from the COVID-19 crisis. Distancing oneself from difficult situations seems to help reduce the negative psychological impact and is a common way people deal with stress and trauma (Mäntymäki et al. 2022; Wang et al. 2022). In the context of ED, this emotional detachment plays a specific role and is often used to cope with life’s challenges (Reid et al. 2020). However, some argue that it can also perpetuate the disorder. In this perspective, detachment has a mixed effect: it may be beneficial for one’s current well-being but could hinder the ability to express emotions. An interesting finding from our study is that after 2 years, ED individuals had a more negative view of the psychological and behavioral effects of the 2020 lockdown compared to HS. This contrast was not observed in the HS group. Although the overall improvement in psychological well-being can be attributed to the treatment received, the negative change in how emotions are framed represents a unique aspect worth noting.

The primary negative effects of the COVID-19 pandemic were associated with the crisis itself and the subsequent disruption of routines, including social isolation and restrictions that limited access to healthcare interventions (Haghshomar et al. 2022). These appear to be the primary factors characterizing the initial deterioration of psychological well-being in ED patients (Monteleone et al. 2021a), even though they overwhelm everyone. Currently, there is no definitive explanation for the increased vulnerability of individuals with ED compared to the general population, although specific factors have been suggested, including changes in the social and home environment, self-isolation, disruption in accessing healthcare, and difficulties in maintaining compensatory behaviors (Fang et al. 2022). Few studies have longitudinally evaluated the effects of the pandemic on the ED population, but the few existing also found improvements in symptomatology over time (Carmassi et al. 2022; Sharpe et al. 2022). Our data corroborate evidence of a significant improvement in psychological burden in individuals, especially people with ED. In fact, both groups reported decreased psychopathological scores in the second evaluation. Individuals with ED reported a more significant reduction in symptoms, even if significant differences with HS remain and could be linked to the presence of the specific psychopathology and the specific burden related to people diagnosed with ED during the pandemic. Interestingly, HS reported reduced concerns about body image and hyperarousal, two constructs related in the general population to fear of COVID-19 and its consequences on people’s lives (Sanchez-Gomez et al. 2021; Snuggs and McGregor 2021).

Conversely, ED patients reported more psychologically severe effects of the COVID-19 crisis after 2 years than after 1 year. This evidence aligns with recent evidence of changes in autobiographical memories over time in individuals with traumatic histories (Booker et al. 2022). In fact, coherence, details, and interpretation could require time, with implications for awareness of symptoms and behaviors that require a specific focus (Booker et al. 2020; Fivush et al. 2017). For these reasons, the psychological effects of the COVID-19 crisis may require some time to be recognized by patients, considering the COVID-19 pandemic as a traumatic event (Monteleone 2021). The confinements, the health insecurity, and the loss of social connections have exposed people to an acute traumatic event that has significantly affected mental health, in terms of emotional regulation, internalization, and eating symptoms. Time could be a specific element that helped emergency department patients improve their well-being, and treatment could have a role. These results corroborate the pandemic as a traumatic event because the data from the trauma literature are similar (Porter and Birt 2001). However, we have also found a more negative evaluation of the 2020 lockdown after 2 years than after 1 year, with a degradation of the memories that could be defined as a reframing bias in recalling a specific event linked to autobiographical memory, which might help to understand the patients’ psychological burden. In fact, individuals with ED present overgeneralized autobiographical memories (Dalgleish et al. 2003; Tenconi et al. 2021), with difficulties in recalling specific positive and adverse events. This aspect can lengthen the time required to structure trauma memories and could be associated with a negative reframing of them. Indeed, a specific adverse effect has been described in people with a traumatic history who reported degradation of their autobiographical memories, and it is called fading affect bias (Ritchie et al. 2006). This bias is more pronounced when social resources are scarce, a characteristic observed in people with ED that also influences treatment outcomes (Southward et al. 2014). Although data on autobiographical memories in EDs is still preliminary, the potential role of specific interventions like imagery rescripting has been discussed, as it may play a role in reducing the negative reframing of memories (Kadriu et al. 2022).

Lastly, another possible explanation is that prolonged exposure to COVID-19 information and public health concerns due to the ongoing pandemic may have allowed for the structure of memories and emotions, which is typically hindered by overgeneralized memories. This phenomenon is common among patients with depression, who tend to overestimate the experience of negative emotions. A negative memory bias (Urban et al. 2018) broadly characterizes a depressogenic cognitive processing style in several psychiatric conditions (Duyser et al. 2020).

As a limitation, the study applied only self-report measures. These might be affected by different biases, including social desirability bias. Another aspect that might be considered is the use of a proximal control sample, whose results are not generalized to the population. A limitation of our first wave was the collection of anonymous data from the general population. Therefore, a second evaluation was not possible. However, this study has the strength to apply a longitudinal approach, increasing knowledge about the effects of the COVID-19 pandemic in the ED field.

To summarize, the current study demonstrated a decrease in psychological distress related to the COVID-19 pandemic among individuals with ED between 2021 and 2022. However, it also highlighted the presence of possible cognitive mechanisms associated with negative reframe bias and overgeneralized autobiographical memories that could contribute to a worsening recollection of past experiences, moments, emotions, and thoughts. These mechanisms may be considered psychological factors influencing the internalization of concerns. To improve the psychological well-being of people, it may be worthwhile to explore specific interventions that aim at improving positive memory recall and reducing emotional reframing within the field of ED.

## Data Availability

The data sets used and analyzed during the current study are available from the corresponding author on a reasonable request.
